# Data science for the scientific life cycle

**DOI:** 10.7554/eLife.43979

**Published:** 2019-03-06

**Authors:** Daphne Ezer, Kirstie Whitaker

**Affiliations:** 1 Alan Turing InstituteLondonUnited Kingdom; 2 Department of StatisticsUniversity of WarwickCoventryUnited Kingdom; 3 Department of PsychiatryUniversity of CambridgeCambridgeUnited Kingdom

**Keywords:** data science, reproducibility, open science, experimental design, None

## Abstract

Data science can be incorporated into every stage of a scientific study. Here we describe how data science can be used to generate hypotheses, to design experiments, to perform experiments, and to analyse data. We also present our vision for how data science techniques will be an integral part of the laboratory of the future.

## Introduction

A key tenet of the scientific method is that we learn from previous work. In principle we observe something about the world and generate a hypothesis. We then design an experiment to test that hypothesis, set up the experiment, collect the data and analyse the results. And when we report our results and interpretation of them in a paper, we make it possible for other researchers to build on our work.

In practice, there are impediments at every step of the process. In particular, our work depends on published research that often does not contain all the information required to reproduce what was reported. There are too many possible experimental parameters to test under our time and budget constraints, so we make decisions that affect how we interpret the outcomes of our experiments. As researchers, we should not be complacent about these obstacles: rather, we should always look towards new technologies, such as data science, to help us improve the quality and efficiency of scientific research.

Data science could easily be dismissed as a simple rebranding of "science" – after all, nearly all scientists analyse data in some form. An alternative definition of a data scientist is someone who develops new computational or statistical analysis techniques that can easily be adapted to a wide range of scenarios, or who can apply these techniques to answer a specific scientific question. While there is no clear dividing line between data science and statistics, data science generally involves larger datasets. Moreover, data scientists often think in terms of training predictive models that can be applied to other datasets, rather than limiting the analysis to an existing dataset.

Data science emerged as a discipline largely because the internet led to the creation of incredibly large datasets (such as ImageNet, a database of 14 million annotated images; Krizhevsky et al., 2012). The availability of these datasets enabled researchers to apply a variety of machine learning algorithms which, in turn, led to the development of new techniques for analysing large datasets. One area in which progress has been rapid is the automated annotation and interpretation of images and texts on the internet, and these techniques are now being applied to other data-rich domains, including genetics and genomics (Libbrecht and Noble, 2015) and the study of gravitational waves (Abbott et al., 2016).

It is clear that data science can inform the analysis of an experiment, either to test a specific hypothesis or to make sense of large datasets that have been collected without a specific hypothesis in mind. What is less obvious, albeit equally important, is how these techniques can improve other aspects of the scientific method, such as the generation of hypotheses and the design of experiments.

Data science is an inherently interdisciplinary approach to science. New experimental techniques have revolutionised biology over the years, from DNA sequencing and microarrays in the past to CRISPR and cryo-EM more recently. Data science differs in that it is not a single technique, but rather a framework for solving a whole range of problems. The potential for data science to answer questions in a range of different disciplines is what excites so many researchers. That said, however, there are social challenges that cannot be fixed with a technical solution, and it is all too easy for expertise to be "lost in translation" when people from different academic backgrounds come together.

**Figure 1.**
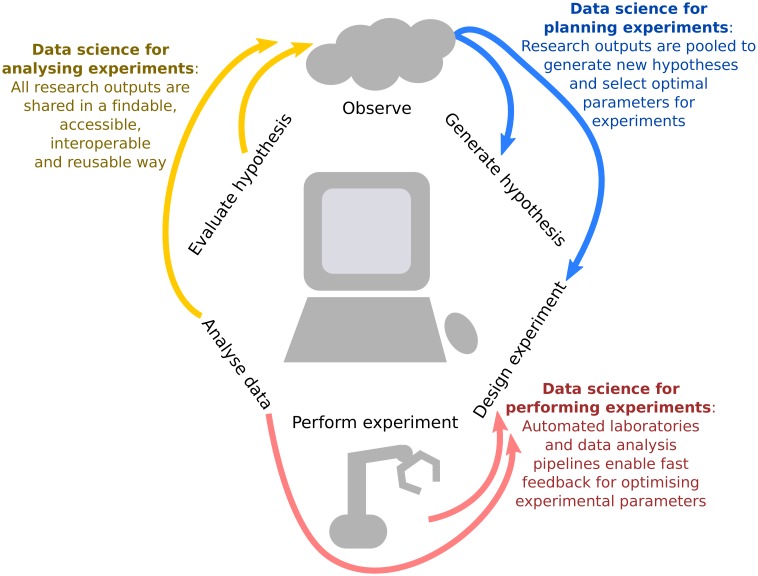
Integrating data science into the scientific life cycle. Data science can be used to generate new hypotheses, optimally design which observations should be collected, automate and provide iterative feedback on this design as data are being observed, reproducibly analyse the information, and share all research outputs in a way that is findable, accessible, interoperable and reusable (FAIR).We propose a virtuous cycle through which experiments can effectively and efficiently "stand on the shoulders" of previous work in order to generate new scientific insights.

In October 2018, we brought together statisticians, experimental researchers, and social scientists who study the behaviour of academics in the lab (and in the wild) at a workshop at the Alan Turing Institute in London to discuss how we can harness the power of data science to make each stage of the scientific life cycle more efficient and effective. Here we summarise the key points that emerged from the workshop, and propose a framework for integrating data science techniques into every part of the research process (Figure 1). Statistical methods can optimise the power of an experiment by selecting which observations should be collected. Robotics and software pipelines can automate data collection and analysis, and incorporate machine learning analyses to adaptively update the experimental design based on incoming data. And the traditional output of research, a static PDF manuscript, can be enhanced to include analysis code and well-documented datasets to make the next iteration of the cycle faster and more efficient. We also highlight several of the challenges, both technical and social, that must be overcome to translate theory into practice, and share our vision for the laboratory of the future.

## Data science for planning experiments

Hypothesis-driven research usually requires a scientist to change an independent variable and measure a dependent variable. However, there are often too many parameters to take account of. In plant science, for instance, these parameters might include temperature, exposure to light, access to water and nutrients, humidity and so on, and the plant might respond to a change in each of these in a context-dependent way.

Data scientists interested in designing optimal experiments must find ways of transforming a scientific question into an optimisation problem. For instance, let us say that a scientist wants to fit a regression model of how temperature and light exposure influence wheat growth. Initially they might measure the height of the wheat at a number of combinations of temperature and light exposure. Then, the scientist could ask: what other combinations of temperature and light exposure should I grow the wheat at in order to improve my ability to predict wheat growth, considering the cost and time constraints of the project?

At the workshop Stefanie Biedermann (University of Southampton) discussed how to transform a wide range of experimental design questions into optimisation problems. She and her colleagues have applied these methods to find optimal ways of selecting parameters for studies of enzyme kinetics (Dette and Biedermann, 2003) and medical applications (Tompsett et al., 2018). Other researchers have used data science to increase the production of a drug while reducing unwanted by-products (Overstall et al., 2018). The process iteratively builds on small number of initial experiments that are conducted with different choices of experimental parameters (such as reagent concentrations, temperature or timing): new experimental parameters are then suggested until the optimal set are identified.

Optimal experimental design can also help researchers fit parameters of dynamical models, which can help them develop a mechanistic understanding of biological systems. Ozgur Akman (University of Exeter) focuses on dynamic models that can explain how gene expression changes over time and where the model parameters represent molecular properties, such as the transcription rates or mRNA degradation rates. As an example he explained how he had used this approach to find the optimal parameters for a mathematical model for the circadian clock (Aitken and Akman, 2013). Akman also described how it is possible to search for possible gene regulatory networks that can explain existing experimental data (Doherty, 2017), and then select new experiments to help distinguish between these alternative hypotheses (Sverchkov and Craven, 2017). For instance, the algorithm might suggest performing a certain gene knockout experiment, followed by RNA-seq, to gain more information about the network structure.

A clear message from the workshop was that statisticians need to be involved in the experimental design process as early as possible, rather than being asked to analyze the data at the end of a project. Involving statisticians before data collection makes it more likely the scientist will be able to answer the research questions they are interested in. Another clear message was that the data, software, infrastructure and the protocols generated during a research project were just as important as the results and interpretations that constitute a scientific paper.

## Data science for performing experiments

In order to effectively plan an experiment, it is necessary to have some preliminary data as a starting point. Moreover, ensuring that the data collected during a particular experiment is used to inform the planning process for future experiments will make the whole process more efficient. For standard molecular biology experiments, this kind of feedback loop can be achieved through laboratory automation.

Ross King (University of Manchester) and co-workers have developed the first robot scientists – laboratory robots that physically perform experiments and use machine learning to generate hypothesis, plan experiments, and perform deductive reasoning to come to scientific conclusions. The first of these robots, Adam, successfully identified the yeast genes encoding orphan enzymes (King et al., 2009), and the second, Eve, intelligently screened drug targets for neglected tropical diseases (Williams et al., 2015). King is convinced that robot scientists improves research productivity, and also helps scientists to develop a better understanding of science as a process (King et al., 2018). For instance, an important step towards building these robotic scientists was the development of a formal language for describing scientific discoveries. Humans might enjoy reading about scientific discoveries that are described in English or some other human language, but such languages are subject to ambiguity and exaggeration. However, translating the deductive logic of research projects into the formal languages of "robotic scientists" should lead to a more precise description of our scientific conclusions (Sparkes et al., 2010).

Let us imagine that a research team observe that plants with a gene knockout are shorter than wild type plants. Their written report of the experiment will state that this gene knockout results in shorter plants. They are likely to leave unsaid the caveat that this result was only observed under their experimental set-up and, therefore, that this may not be the case under all possible experimental parameters. The mutant might be taller than a wild type plant under certain lighting conditions or temperatures that were not tested in the original study. In the future, researchers may be able to write their research outcomes in an unambiguous way so that it is clear that the evidence came from a very specific experimental set-up. The work done to communicate these conditions in a computer-readable format will benefit the human scientists who extend and replicate the original work.

Even though laboratory automation technology has existed for a number of years, it has yet to be widely incorporated into academic research environments. Laboratory automation is full of complex hardware that is difficult to use, but a few start-ups are beginning to build tools to help researchers communicate with their laboratory robots more effectively. Vishal Sanchania (Synthace) discussed how their software tool Antha enables scientists to easily develop workflows for controlling laboratory automation. Furthermore, these workflows can be iterative: that is, data collected by the laboratory robots can be used within the workflow to plan the next experimental procedure (Fell et al., 2018).

One benefit of having robotic platforms perform experiments as a service is that researchers are able to publish their experimental protocols as executable code, which any other researcher, from anywhere around the world, can run on another automated laboratory system, improving the reproducibility of experiments.

## Data science for reproducible data analysis

As the robot scientists (and their creators) realised, there is a lot more information that must be captured and shared for another researcher to reproduce an experiment. It is important that data collection and its analysis are reproducible. All too often, there is no way to verify the results in published papers because the reader does not have access to the data, nor to the information needed to repeat the same, often complex, analyses (Ioannidis et al., 2014). At our workshop Rachael Ainsworth (University of Manchester) highlighted Peng’s description of the reproducibility spectrum, which ranges from “publication only” to "full replication" with linked and executable code and data (Peng, 2011). Software engineering tools and techniques that are commonly applied in data science projects can nudge researchers towards the full replication end of the spectrum. These tools include interactive notebooks (Jupyter, Rmarkdown), version control and collaboration tools (git, GitHub, GitLab), package managers and containers to capture computational environments (Conda, Docker), and workflows to test and continuously integrate updates to the project (Travis CI). See Beaulieu-Jones and Greene, 2017 for an overview of how to repurpose these tools for scientific analyses.

Imaging has always been a critical technology for cell and developmental biology (Burel et al., 2015), ever since scientists looked at samples through a microscope and made drawings of what they saw. Photography came next, followed by digital image capture and analysis. Sébastien Besson (University of Dundee) presented a candidate for the next technology in this series, a set of open-source software and format standards called the Open Microscopy Environment (OME). This technology has already supported projects as diverse as the development a deep learning classifier to identify patients with clinical heart failure (Nirschl et al., 2018), to the generation of ultra-large high resolution electron microscopy maps in human, mouse and zebrafish tissue (Faas et al., 2012).

The OME project also subscribes to the philosophy that data must be FAIR: findable, accessible, interoperable and reusable (Wilkinson et al., 2016). It does this as follows: i) data are made *findable* by hosting them online and providing links to the papers the data have been used in; ii) data are made *accessible* through an open API (application programming interface) and the availability of highly curated metadata; iii) data are made *interoperable* via the Bio-Formats software, which allows more than 150 proprietary imaging file formats to be converted into a variety of open formats using a common vocabulary (Linkert et al., 2010); iv) data, software and other outputs are made *reusable* under permissive open licences or through "copyleft" licences which require the user to release anything they derive from the resource under the same open licence. (Alternatively, companies can pay for private access through Glencoe Software which provides a commercially licenced version of the OME suite of tools.).

Each group who upload data to be shared through OME's Image Data Resource can choose their own license for sharing their data, although they are strongly encouraged to use the most open of the creative commons licenses (CC-BY or CC0). When shared in this way, these resources open up new avenues for replication and verification studies, methods development, and exploratory work that leads to the generation of new hypotheses.

## Data science for hypothesis generation

A hypothesis is essentially an "educated guess" by a researcher about what they think will happen when they do an experiment. A new hypothesis usually comes from theoretical models or from a desire to extend previously published experimental research. However, the traditional process of hypothesis generation is limited by the amount knowledge an individual researcher can hold in their head and the number of papers they can read each year, and it is also susceptible to their personal biases (van Helden, 2013).

In contrast, machine learning techniques such as text mining of published abstracts or electronic health records (Oquendo et al., 2012), or exploratory meta-analyses of datasets pooled from laboratories around the world, can be used for automated, reproducible and transparent *hypothesis generation*. For example, teams at IBM Research have mined 100,000 academic papers to identify new protein kinases that interact with a protein tumour suppressor (Spangler, 2014) and predicted hospital re-admissions from the electronic health records of 5,000 patients (Xiao et al., 2018). However, the availability of datasets, ideally datasets that are FAIR, is a prerequisite for automated hypothesis generation (Hall and Pesenti, 2017).

## The challenges of translating theory into practice

When we use data science techniques to design and analyse experiments, we need to ensure that the techniques we use are transparent and interpretable. And when we use robot scientists to design, perform and analyse experiments, we need to ensure that science continues to explore a broad range of scientific questions. Other challenges include avoiding positive feedback loops and algorithmic bias, equipping scientists with the skills they need to thrive in this new multidisciplinary environment, and ensuring that scientists in the global south are not left behind. We discuss all these points in more detail below.

### Interpreting experimental outcomes

When data science is used to make decisions about *how* to perform an experiment, we need to ensure that scientists calibrate their level of trust appropriately. On one hand, biologists will need to relinquish some level of control and to trust the computer program to make important decisions for them. On the other hand, we must make sure that scientists do not trust the algorithms so much that they stop thinking critically about the outcomes of their experiments and end up misinterpreting their results. We also want to ensure that scientists remain creative and open to serendipitous discoveries.

We discussed above the importance of having a formal (machine-readable) language that can be used to describe both scientific ideas and experimental protocols to robots. However, it is equally important that the results and conclusions of these experiments are expressed in a human-understandable format. Ultimately, the goal of science is not just to be able to predict natural phenomenon, but also to give humans a deeper insight into the mechanisms driving the observed processes. Some machine learning methods, such as deep learning, while excellent for predicting an outcome, suffer from a lack of interpretability (Angermueller et al., 2016). How to balance predictability and interpretability for the human reader is an open question in machine learning.

### Positive feedback loops and algorithmic bias

As with all applications of data science to new disciplines, there are risks related to algorithmic bias (Hajian et al., 2016). Recently there have been some concerns over algorithmic bias related to face-recognition of criminals – the face-recognition software was more likely to report a false-positive of a black face than a white face due to biases in the dataset that the software was trained on (Snow, 2017; Buolamwini and Gebru, 2018). Societal parameters shape the input data that is fed into machine learning models, and if actions are taken on the basis of their output, these societal biases will only be amplified.

There are parallel issues with data science for experimental biology – for instance there are certain research questions that are popular within a community through accidents of history. Many people study model organisms such as roundworms and fruit flies because early genetics researchers studied them, and now there are more experimental tools that have been tried and tested on them – a positive feedback loop (Stoeger et al., 2018).

We need to be careful to ensure that any attempt to design experiments has the correct balance between exploring new research ideas and exploiting the existing data and experimental tools available in well-established sub-disciplines.

### Implementation and training

According to Chris Mellingwood (University of Edinburgh), some biologists are *amphibious* and fluidly move between "wet" laboratories and "dry" computing (Mellingwood, 2017). However, many biologists do not know how to code or do not have the required mathematical background to be able to reframe their research questions as data science problems, so it may be difficult for biologists to find ways of using these new tools to design experiments in their own laboratories. They might not even realise that there is a tool available to help them resolve the experimental design problems that they face. Researchers may need specialised training in order to learn how to interact with data science tools in a efficient and effective way.

Reproducible data analysis alone requires an understanding of version control, at least one, if not multiple, programming languages, techniques such as testing, containerisation and continuous integration. Machine learning and optimisation algorithms require detailed statistical knowledge along with the technical expertise – sometimes including high performance computing skills – to implement them. Requiring all these skills along with the robotics engineering expertise to build an automated lab is outside of the capacity of most researchers trained by the current system.

### Infrastructure and accessibility

Even once a system is built, it needs to be constantly adapted as science progresses. There is a risk that by the time a platform is developed, it might be out of date. Sarah Abel (University of Iceland) discussed how university incentive systems do not always reward the types of activities that would be required for incorporating data science into a laboratory, such as interdisciplinary collaborations or maintenance of long-term infrastructure.

Furthermore, due to the burden of developing and maintaining the infrastructure needed for this new approach to science, some researchers may be left behind. Louise Bezuidenhout (University of Oxford) explained that even though one of the goals of "data science for experimental design" is to have open and "accessible" data available around the world, scientists in the global south might not have access to computing resources needed for this approach (Bezuidenhout et al., 2017). Therefore, we need to consider how the benefits of data science and laboratory automation techniques are felt around the world.

### Augmentation, not automation

As we discuss the role of data science in the cycle of research, we need to be aware that these technologies should be used to augment, not replace, human researchers. These new tools will release researchers from the tasks that a machine *can* do well, giving them time and space to work on the tasks that only humans can do. Humans are able think "out-of-the-box", while the behaviour of any algorithm will inherently be restricted by its code.

Perhaps the last person you might imagine supporting the integration of artificial and human intelligence is Garry Kasparov, chess grand master. Kasparov lost to the IBM supercomputer Deep Blue in 1997 but more than 20 years later he is optimistic about the potential for machines to provide insights into how humans see the world (Kasparov, 2017). An example that is closer to the life sciences is the citizen science game *BrainDr*, in which participants quickly assess the quality of brain imaging scans by swiping left or right (Keshavan et al., 2018). Over time, there were enough ratings to train an algorithm to automatically assess the quality of the images. The tool saves researchers thousands of hours, permits the quality assessment of very large datasets, improves the reliability of the results, and is really fun!

So where does this leave the biologist of the future? Experimentalists can continue to do creative research by, for example, designing new protocols that enable them to study new phenomena and measure new variables. There are also many experimental protocols that are performed so rarely that it would be inefficient to automate them. However, humans will not need to carry out standard protocols, using as purifying DNA, but they might still need to know how to perform various specialised tasks, such as dissecting specimens. They will also need to constantly update the robotic platform to incorporate new experimental protocols.

Finally and most crucially, the biologist of the future will need to provide feedback into the cycle of research – providing insight into what hypotheses are interesting to the community, thinking deeply about how experimental results fit into the theories proposed by the broader community, and finding innovative connections across disciplinary boundaries. Essentially, they will be focused on the varied and interesting parts of science, rather than the mundane and repetitive parts.

Box 1. What can you do now?

**Planning an experiment**

In the lab of the future, we envision that experimental parameters will be chosen in a theoretically sound way, rather than through *ad hoc* human decision making. There are already plenty of tools to help researchers plan their experiments, including tools for selecting optimal time points for conducting an experiment (Kleyman et al., 2017; Ezer and Keir, 2018), a collection of R packages that enable optimisation of experimental design (CRAN) and the acebayes package, which takes prior information about the system as input, and then designs experiments that are most likely to produce the best outputs (Overstall et al., 2017).

**Performing an experiment**

In the future, standard molecular biology experiments will be performed by robots, and executable experimental protocols will be published alongside each journal article to ensure reproducibility. Although many labs do not have access to laboratory automation, there are many associated techniques that will improve the reproducibility of research. For instance, systems like Protocols.io can help researchers to describe protocols in unambiguous ways that can be easily understood by other researchers. Sharing laboratory know-how, using tools such as OpenWetWare, will also enable a tighter feedback look between performing and planning experiments.

**Analysing experimental data**

In a few years, we hope that many more data formats and pipelines will be standardised, reproducible and open-access. For researchers who are most comfortable in a wet-lab environment, the article "Five selfish reasons to work reproducibly" makes a strong case for learning how to code (Markowetz, 2015). Jupyter notebooks are an easy way to share data analyses with embedded text descriptions, executable code snippets, and figures. Workshops run by The Carpentries are a good way to learn software skills such as the unix shell, version control with git, and programming in Python or R or domain specific techniques for data wrangling, analysis and visualisation.

**Sharing your work**

For anyone keen to know more about archiving their data, preprints, open access and the pre-registration of studies, we recommend the "Rainbow of Open Science Practices" (Kramer and Bosman, 2018) and Rachael Ainsworth's slides from our workshop (Ainsworth, 2018).

**Generating new hypotheses**

Pooling studies across laboratories and textual analysis of publications will help identify scientific questions worth studying. The beginning of a new cycle of research might start with an automated search of the literature for similar research (Extance, 2018) with tools such as Semantic Scholar from the Allen Institute for Artificial Intelligence. Alternatively, you could search for new data to investigate using Google's Dataset Search, or more specific resources from the European Bioinformatics Institute or National Institute of Mental Health Data Archive.
